# Dendritic Cell and Cancer Therapy

**DOI:** 10.3390/ijms24044253

**Published:** 2023-02-20

**Authors:** Domenico Galati, Serena Zanotta

**Affiliations:** Hematology-Oncology and Stem Cell Transplantation Unit, Department of Hematology and Innovative Diagnostic, Istituto Nazionale Tumori-IRCCS-Fondazione “G. Pascale” Napoli, 80131 Napoli, Italy

Dendritic cells (DCs) are acknowledged as the most potent professional antigen-presenting cells (APCs), able to induce adaptive immunity and support the innate immune response. In this regard, DCs represent a promising means for cancer vaccines to strengthen the anti-cancer immune response to overcome the immunosuppressive tumor microenvironment. Nevertheless, cancers can avoid anti-tumor immunity by expressing immune inhibitory molecules and secreting suppressive cytokine-inactivating DCs and effector T lymphocytes [1].

As a result, the safety and feasibility of DC-based immunotherapies in the treatment of cancer tumors are well-documented. The clinical benefits are suboptimal and still unsatisfactory despite the induction and strengthening of specific anti-tumor immune responses [1]. Therefore, new insights will help define molecules and pathways that can modulate the performance of DC subsets. This Special Issue of the *International Journal of Medical Sciences* focuses on recent advances in this field. A better understanding of how DCs induce, regulate, and maintain T cell immunity will undoubtedly help to improve the immunotherapeutic efficacy of the current and future approaches for tumor disease control. 

With specific reference to this research topic, Zhang et al. [2] studied the role of intranasal administration of ecklonia cava-extracted fucoidan (ECF) on the induction of anti-cancer responses in the respiratory mucosal immune system. ECF is a mucosal adjuvant, used to formulate mucosal vaccines that can boost the immune response against targeted diseases. Notably, intranasal administration of ECF stimulated the activation of DCs, natural killer (NK) cells, and T cells in the mediastinal lymph nodes of C57BL/6 and BALB/c mice. Moreover, intranasal injection of ECF enhanced the anti-PD-L1 antibody-induced anti-cancer activities against B16 melanoma and CT-26 carcinoma tumor growth in mice lungs. This study is particularly intriguing because the upregulation of costimulatory and MHC molecules and the blockade of PD-L1 in DCs could improve the induction of T-cell-mediated anti-cancer immunity. Wang et al. [3] likewise examined the effect of intranasal administration of Codium fragile polysaccharides (CFPs) in mice. CFPs are a natural polysaccharide that have a promising impact on animal immunity regulation. Intranasal administration of CFPs can activate DCs, macrophages, T lymphocytes, and NK cells in the mediastinal lymph node (mLN), contributing to anti-cancer immune responses against the growth of Lewis lung carcinoma. In particular, conventional DCs (cDCs) were increased in mLNs by the upregulation of C-C motif chemokine receptor 7 (CCR7) expression. Notably, the combinatorial approach of CFPs with anti-programmed cell death-ligand 1 (PD-L1) antibody (Ab) improved the therapeutic effect of anti-PD-L1 Ab against lung cancer. Therefore, the intranasal administration of CFP can elicit immunity against lung cancer in mice.

Moreover, Han et al. [4] have first reported the immunological functions of extracellular vesicles (EV) derived from an edible plant, Petasites japonicus (PJ), that can induce maturation and activation of DCs. Notably, PJ-EVs can be potent immunostimulatory candidates, since PJ-EV-treated DCs produce increasing amounts of IL-12, the key factor supporting T helper 1 polarization and the cytotoxic T lymphocyte response, which could have potent anti-cancer effects. Successively, Nava et al. [5] have examined the literature data and recent advances in clinical studies examining DCs. Their detailed review further confirms that combinatorial approaches (chemotherapy and radiotherapy) can improve the efficacy of DC-based vaccines in cancer treatment. Accordingly, Verheye et al. [6] have described the well-known DC deficiencies in multiple myeloma (MM) patients, summarizing the DC-based vaccination protocols adopted in MM. Furthermore, they highlighted that tissue-derived DC subsets could give rise to next-generation DC vaccination for MM with superior features for DC-based immunotherapy. Interestingly, DC-based vaccines, in combination with therapies targeting the suppressive microenvironment (e.g., immunomodulatory imide drugs (IMiDs) and immune checkpoint inhibitors (ICIs)) and other treatments such as chemotherapy, could help improve anti-tumor immune responses and the clinical outcome in MM patients.

Kang et al. [7] also stressed that a thorough understanding of the characteristics of tumors and DCs could increase the efficacy of DC-based cancer immunotherapy. DCs are diverse, and cancers can be characterized as hot or cold by their immunogenicity, which makes targeting them challenging. Consequently, DC-based therapies are more efficient in highly immunogenic hot tumors than poorly immunogenic cold tumors. In particular, the priming method can convert a hard tumor microenvironment to a hot tumor microenvironment and increase DC vaccine efficacy. Lastly, Rackl et al. [8] investigated whether stimulating immune cells in DCs of acute myeloid leukemia (AML) patients and healthy donors leads to increased leukemia-specific integrin beta-7 (ITGβ7) expression in immune cells. ITGβ7 is a subunit of the integrin receptor expressed on the surface of immune cells, mediating cell–cell adhesions, and is involved in an anti-tumor immune response. The results demonstrated that DC-based immune therapies could significantly stimulate the immune system against leukemic blasts by increasing the percentages of (leukemia-specific) ITGβ7-expressing immune cells. In this context, ITGβ7 may become a predictor for successful AML and myelodysplastic syndrome (MDS) therapies.

All the manuscripts in this Special Issue have critically underlined the DC vaccine strengths, while not understating the relevant unresolved questions about the suboptimal and still unsatisfactory clinical benefits of current DC-based cancer treatments. Even though this Special Issue is not an exhaustive list of the studies in this field, we believe that each manuscript published here will contribute to the development of new, more effective DC-based cancer immunotherapies.

## Data Availability

Not applicable.
